# Hydrodynamic ultrasonic maxillary sinus lift: Review of a new technique and presentation of a clinical case

**DOI:** 10.4317/medoral.17430

**Published:** 2011-12-06

**Authors:** Rocío Velázquez-Cayón, Manuel M. Romero-Ruiz, Daniel Torres-Lagares, Beatriz Pérez-Dorao, Marcel Wainwright, Camilo Abalos-Labruzzi, José L. Gutiérrez-Pérez

**Affiliations:** 1 Professor of Master in Oral Surgery. University of Seville; 2 Professor of Oral Surgery. Coordinator of Master’s in Oral Surgery at the University of Seville; 3 Professor on Odontologic Materials. University of Seville; 4 Head of the Oral and Maxillofacial Surgery Department from the Hospital Universitario Virgen del Rocío, on temporary leave. Professor of Oral Surgery. Head of Master’s in Oral Surgery at the University of Seville

## Abstract

Objectives: Placing implants in the posterior maxillary area has the drawback of working with scarce, poor quality bone in a significant percentage of cases. Numerous advanced surgical techniques have been developed to overcome the difficulties associated with these limitations. Subsequent to reports on the elevation of the maxillary sinus through the lateral approach, there were reports on the use of the crestal approach, which is less aggressive but requires a minimal amount of bone. Furthermore, it is more sensitive to operator technique, as the integrity of the sinus membrane is checked indirectly. The aim of this paper is to review the technical literature on minimally invasive sinus lift and compare the advantages of different techniques with Intralift™, a new technique. Study Design: The present study is a review of techniques used to perform minimally invasive sinus lift published in Cochrane, Embase and Medline over the past ten years and the description of the crestal sinus lift technique based on minimally invasive piezosurgery, with the example of a case report. Results: Only eight articles were found on minimally invasive techniques for sinus lift. The main advantage of this new technique, Intralift, is that it does not require a minimum amount of crestal bone (indeed, the smaller the width of the crestal bone, the better this technique is performed). The possibility of damage to the sinus membrane is minimised by using ultrasound based hydrodynamic pressure to lift it, while applying a very non-aggressive crestal approach. Conclusions: We believe that this technique is an advance in the search for less traumatic and aggressive techniques, which is the hallmark of current surgery.

** Key words:** Sinus lift, surgical technique, minimally invasive surgery, ultrasound surgery.

## Introduction

 An essential condition for success in dental implant treatment is the amount and quality of bone in the area on which we decide to place the implant after careful diagnosis and planning. The posterior area of the upper jaw has anatomical features that make it unique compared to other areas, mainly due to the presence of the maxillary sinus (1). After tooth loss there is progressive bone resorption, combined with sinus pneumatisation and loss of bone height and quality, which greatly hinder the placement of dental implants (2). 

To overcome this loss of bone height, sinus lift techniques have been used (Boyne and James) (3), which increase the availability of bone in the posterior maxilla and thus achieve successful implant treatment. Tatum (4) subsequently developed a sinus lift via a lateral approach with osteotomy of the vestibular cortex, so that the space gained after raising the membrane was filled with augmentation material that would maintain a space for the time necessary for the bone defect to be filled by the subject’s own bone material. Different graft materials with autologous bone as a benchmark have been studied successively by different authors. Esposito et al., in a review conducted within the Cochrane Collaboration organisation concluded that bone substitutes, Bio-Oss™ (Geistlich Biomaterials, Germany) or Cerasorb™(Curasan AG, Germany) could be used to replace autologous bone in sinus lift procedures in cases of extremely atrophic sinuses (5). 

Another step in the search for less invasive techniques was the use of compressive osteotomes (Summers) to lift the sinus membrane with a closed technique using a crestal approach (6,7), and additional filling of the sinus with different graft materials. Soltan and Smiler (8) proposed a balloon technique (Antral Membrane Balloon Elevation, AMBE), consisting in gently detaching the membrane using a latex balloon inflated with saline solution. This technique offers advantages such as reduced postoperative pain, bleeding and wound infection rates. 

An important contribution to oral surgery in general and the development of such techniques in particular, was the introduction of piezoelectric surgery. Thus, Torrella et al. (9) proposed the use of piezoelectric surgery for lateral osteotomies. They are performed with a bone preserving incision so they are less traumatic and reduce the risk of perforation of the Schneiderian membrane, and achieve better view during surgery. Based on the use of piezoelectric surgery attempts have been made to simplify the sinus lift technique to offer patients an intervention as atraumatic as possible, with milder postoperative discomfort. To this end, Troedhan, Kurrek, Wainwright and Jank (10) in conjunction with the Acteon Group (France) have developed the Intralif™ (Acteon Satelec, France) technique. A minimally invasive technique for lifting the maxillary sinus floor using piezoelectric surgery based on a specific set of tips for the application of ultrasound. This technique opens a wide range of possibilities in terms of reducing the complexity and morbidity of open sinus lift. 

The aim of this paper is a review of the literature on the Intralift ™ technique, with descriptions and case reports to illustrate and assess this technique. 

## Material and Method 

To carry out this review we focused our search on articles published in the last 10 years, indexed in Cochrane, Embase and Medline, using the Pubmed search engine in this last case. The search terms were: sinus lift, maxillary sinus lift, sinus lift technique, minimally invasive sinus lift. 

We also carried out a manual search of the following journals available in the Health Library of the Faculty of Dentistry (Universidad de Sevilla): The Journal of Craniofacial Surgery, The International Journal of Prosthodontics, Oral Surgery Oral Medicine Oral Pathology Oral Radiology and Endodontics, Journal of Oral Maxillofacial Surgery, Implant Dentistry, Journal of Oral Surgery, Dental Clinics of North America, Compendium of Continuing Education in Dentistry, Journal of Esthetic Dentistry, International Journal of Oral and Maxillofacial Implants and Cochrane Database Systematic Review. 

**Figure 1:**
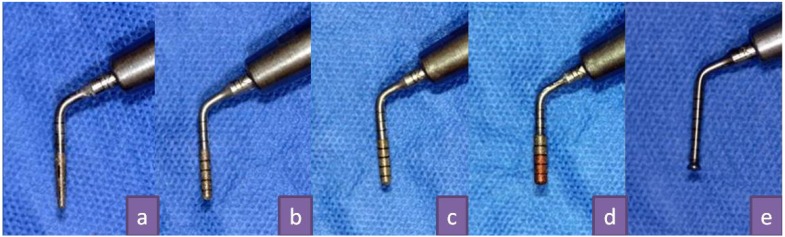
(A) TKW1 tip, (b) TKW2 tip, (c) TKW3 tip, (d) TKW4 tip, (e) TKW5 tip, also called 'Trumpet'.

Regarding the technique presented in this paper, the preparation of the hole to access the floor of the sinus floor is performed at the crest, sequencing the Intralift™ system of pints (Fig. 1) and the Piezotome – Implant Center 2™ (Acteon Satelec, France), to control the vibration of the tips and their irrigation. The Piezotome makes it possible to work with these ultrasonic tips with four power modes D-1 to D-4, which correspond to the classification of bone quality (1 = dense bone, 4 = very spongy bone). D-1, D-2 power is used at the beginning, for cortical bone, and D-3, D-4 at the end of the procedure for cancellous bone and to lift the sinus membrane. 

At this point it is necessary to follow the manufacturer’s instructions, using the following drill sequences: 

1. ‘Pilot drilling’; a conical diamond tip (TKW 1 - Ø 1.35mm) is used in D2 mode, with irrigation of 70-100 ml/min (Fig. 1). 

2. ‘Preliminary drilling’: a cylindrical diamond tip (TKW 2-Ø 2.1mm) is used in D2-D3 mode with an irrigation of 70-100 ml/min (Fig. 1). 

3. ‘Preliminary drilling’: a cylindrical diamond tip (TKW 3 - Ø 2.35mm) is used in a D2-D3 mode with irrigation of 70-100 ml/min (Fig. 1). 

4. ‘Secondary drilling’: a cylindrical diamond tip (TKW 4 - Ø 2.80mm) is used in D2-D3 mode with an irrigation of 70-100 ml/min (Fig. 1). 

5. ‘Trumpet’ a non-diamond tip (TKW 5) (Fig. 1) is used. This is a non-cutting tip, which sprays sterile irrigation causing internal sinus membrane elevation by microcavitation. It is used in D3/D4 mode with an irrigation of 30 - 40 ml/min. It can also be used in non-activated mode to compact material, using it only as a manual instrument. The tip should never be placed in direct contact with the Schneiderian membrane, therefore, haemostatic collagen sponges should be inserted for protection. 

The first 4 drills are only used for widening the preparation, and drill 5 (trumpet) is the only one that really elevates the Schneiderian membrane. 

## Results

As a result of our search we only found eight articles on sinus lift using minimally invasive surgery. All of them have been cited in the literature and included in the discussion to compare these techniques with the one presented. With regard to the clinical application of the technique described in the ‘Material and Method’ section, this is illustrated by the following clinical case. 

 Case Report 

**Figure 2:**
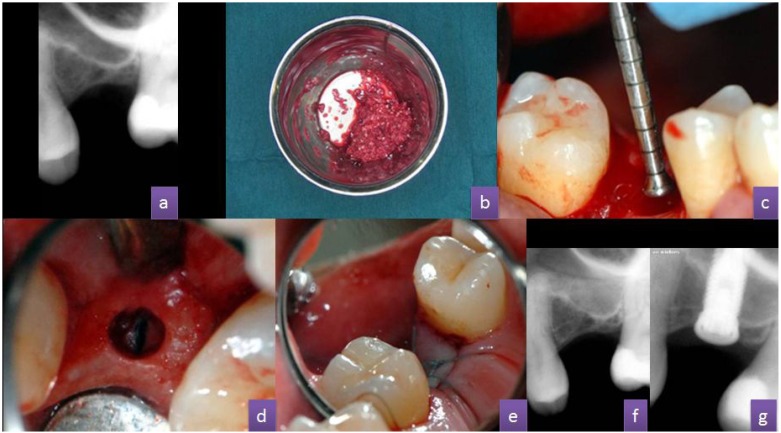
(A) Preoperative periapical X-ray, (b) Mixing blood collected from the edges of the incision with augmentation material (Biogen™, Bioteck, Italy) (c) TKW5 tip, also called 'Trumpet', (d) Hole created on the ridge for subsequent use of a TKW5 tip, (e) 4/0 silicone-coated polyester suture, (f) Periapical X-ray of maxillary sinus with augmentation material inserted, (g) Periapical X-ray of the maxillary sinus with augmentation material and implant in place.

We report the case of a female patient aged 48, without any previous history of note. Insertion of an implant in the 2.6 region was indicated to rehabilitate the edentulous space. The bone was 3-4 mm high in this area (Fig. 2). In most protocols, this case would be treated using a sinus lift technique with a lateral approach.

The technique was carried out under local anaesthetic infiltration in the posterior part of the vestibule and palate. An intrasulcular incision was made without releasing incisions, and then we collected the blood from the edges of the incision using a syringe. This blood was mixed with the filler material before it could clot (Fig. 2). Another possibility is to hydrate the graft material with saline. During the next step we carried out mucoperiosteal detachment. 

We started the preparation with the first drill in the surgical box for implants, to open up the field faster, milling up to 1 mm below the sinus floor, and we then continued work with the ultrasound tips (TKW1 - TKW4), to reach the sinus and broaden the preparation (if we found a subantral bone height of less than 4 mm, and we would begin osteotomy directly with the ultrasound tips). 

After preparing the hole and detaching the Schneiderian membrane, we put in place the first portion of augmentation material (Fig. 2) using the non-activated TKW5 (trumpet) tip (Fig. 2). The progressive introduction of material can cause material conden-sation at the osteotomy entry, so the TKW5 tip must be activated at the weakest power (D4), the lowest possible irrigation volume (10 ml/min), and only for 1 second. This is repeated 2-3 times, in order to propel the material into the space created under the Schneiderian membrane (“Plug and Spray” technique). 

After all the filling material was put in place we used a collagen membrane to cover the hole, prior to the replacement and suturing of the flap (Fig. 2). After surgery we waited between five and eight months before performing the final implant placement (Fig. 2). 

## Discussion

It is possible to find descriptions of many techniques for sinus lift in atrophic maxillae cases in the literature, and most of them are invasive or aggressive. However, the current trend in medicine, and especially in dentistry, is to promote the use of less invasive techniques (minimally invasive surgery). For this reason new sinus lift procedures following this line of thought have been developed and reported. 

Thus, in 2006, Halpern, Halpern et al. (11) published a modification of the Summers’ technique in which during one surgical intervention they placed the implants in ridges of 3 to 4 mm in height. These authors emphasised the need for using a punch for implant placement. Among the variations of the sinus lift technique using compressive osteotomes, Tilotta et al. (12) performed an anatomical study on cadavers, describing a technique for maxillary sinus lift using trephines and osteotomes with stops. With this technique they obtained a sinus lift of 4-6 mm in sinuses that had 5 mm of bottom ridge, with a very low rate of rupture of the sinus membrane. 

Kfir et al. (13) proposed a variation of the Soltan and Smiler balloon technique (AMBE) in 2006. It was called MIAMBE (Minimally Invasive Antral Membrane Balloon Elevation) technique in which the balloon is introduced through the bed prepared for the implant using a crestal approach. The balloon is inflated sequentially to an elevation below 10 mm, and the augmentation material is then introduced and the implant is put in place if it is considered that there is acceptable primary stability. 

In all these techniques with reduced visibility, the surgeon’s experience is a key factor for successful treatment. Furthermore, careful planning of the intervention and a right selection of each case are essential when carrying out these procedures. What is no longer in doubt, in light of published evidence, is the advantage of minimally invasive surgery (14). 

The technique described in this paper has many advantages. Simplifying the procedure by using piezoelectric surgery tips minimises the risk of introducing instruments into the sinus cavity, and by using ultrasound cavitation patient discomfort is reduced, there is no osteotome hammering or lifting of large flaps. The use of ultrasound to perform these surgeries means that we can carry out less traumatic and conservative bone incisions, reducing bleeding and achieving better visibility during surgery. In addition there is a reduction in the risk of perforation of the Schneiderian membrane, since accidental instrument contact with the membrane, as the instruments used are less aggressive than rotary instruments, carries less risk of injury. This problem can be controlled at all times and membrane integrity can be checked using the Valsalva manoeuvre. Even if small perforations are caused, surgery can be completed using a small collagen sponge or collagen membrane to close them. 

Compared with other techniques which use rotary instruments, and minimally invasive techniques such as Summers’ technique, we think that the Intralift™ technique is safer and poses less risk of tissue injury (membranes, arteries ...) Furthermore, by using the cavitation effect there is no need for bone debridement in the surgical site, so there is no risk of bone fragments entering the cavity. 

This technique proved more effective in achieving greater and more homogeneous membrane elevation. Regeneration material is seen very distal and mesial to the preparation. Furthermore, the incision does not depend on the force exerted by the surgeon, the ultrasound does all the work. While the membrane undergoes tensile forces when the balloon technique or Summers’ technique are used, when the Intralift™ technique is used there is no traction because microcavitation gently detaches the membrane in all directions, not just at a pressure point. 

Minimum aggression means that there is less postoperative discomfort, reducing the postoperative pain (minimal use of analgesics), bleeding and wound infection rates. Therefore we think that the minimally invasive Intralift™ maxillary sinus lift technique described can be an effective minimally invasive alternative for atraumatic elevation of the sinus, and therefore must be considered as a possibility which could replace other more aggressive techniques.

However, the lack of comparative clinical trials and the absence of long-term monitoring mean that it is necessary to be cautious and wait for further studies to support the excellent clinical performance seen so far. We also consider that this is an operator sensitive technique and therefore requires a learning curve to achieve its maximum potential and avoid unnecessary damage to patients. 
